# Association between lipid profile and clinical outcomes in COVID-19 patients

**DOI:** 10.1038/s41598-024-62899-y

**Published:** 2024-05-27

**Authors:** Luis Antonio Ochoa-Ramírez, Alberto Kousuke De la Herrán Arita, Jorge Guillermo Sanchez-Zazueta, Efrén Ríos-Burgueño, Joel Murillo-Llanes, Luis Adrián De Jesús-González, Carlos Noe Farfan-Morales, Carlos Daniel Cordero-Rivera, Rosa María del Ángel, Alejandra Romero-Utrilla, Josué Camberos-Barraza, Marco Antonio Valdez-Flores, Alejandro Camacho-Zamora, José Candelario Batiz-Beltrán, Carla Angulo-Rojo, Alma Marlene Guadrón-Llanos, Verónica Judith Picos-Cárdenas, Claudia Desiree Norzagaray-Valenzuela, Ángel Radamés Rábago-Monzón, Jesús Salvador Velarde-Félix, José Manuel Reyes-Ruiz, Juan Fidel Osuna-Ramos

**Affiliations:** 1 Hospital General de Culiacán “Bernardo J. Gastelum”, Servicios de Salud de Sinaloa, Culiacán, Sinaloa Mexico; 2 Facultad de Medicina, Universidad Autónoma de Sinaloa, Culiacán, Sinaloa Mexico; 3 Facultad de Biología, Universidad Autónoma de Sinaloa, Culiacán, Sinaloa Mexico; 4 Departamento de Anatomía Patológica, Centro de Investigación y Docencia en Ciencias de la Salud, Universidad Autónoma de Sinaloa, Hospital Civil de Culiacán, Culiacán, Mexico; 5 Departamento de Investigación del Hospital de la Mujer, Servicios de Salud de Sinaloa, Culiacán, Sinaloa Mexico; 6 Unidad de Investigación Biomédica de Zacatecas, Instituto Mexicano del Seguro Social, Zacatecas, Zacatecas Mexico; 7 Departamento de Ciencias Naturales, Universidad Autónoma Metropolitana, Unidad Cuajimalpa, Ciudad de México, Mexico; 8 Departamento de Infectómica y Patogénesis Molecular, Centro de Investigación y de Estudios Avanzados del Instituto politécnico Nacional, Ciudad de México, Mexico; 9 Departamento de Anatomía Patológica, Instituto Mexicano del Seguro Social, Culiacán, Sinaloa Mexico; 10 Departamento de Investigación en Salud, Unidad Médica de Alta Especialidad, Hospital de Especialidades No. 14, Centro Médico Nacional “Adolfo Ruiz Cortines”, Instituto Mexicano del Seguro Social (IMSS), Veracruz, Mexico; 11 Facultad de Medicina, Región Veracruz, Universidad Veracruzana (UV), Veracruz, Mexico

**Keywords:** SARS-CoV-2, COVID-19 severity, HDL cholesterol, Biomarker, Microbiology, Predictive markers

## Abstract

High-density lipoprotein cholesterol (HDL-c) removes cholesterol, an essential component in lipid rafts, and this cholesterol removal can regulate protein attachment to lipid rafts, modulating their functionality in the immune cell response. Although severe acute respiratory syndrome coronavirus 2 (SARS-CoV-2) infection can alter the lipid profile, there is little information on the role of HDL-c and other lipids in prognostic of the coronavirus disease 2019 (COVID-19) in Mexican population. This study aims to evaluate the predictive value of HDL-c and lipid profile on severity and survival of 102 patients infected with SARS-CoV-2 during the COVID-19 first wave. Our findings, derived from univariate and multivariate Cox proportional hazards regression models, highlighted age and hypertension as significant predictors of survival (HR = 1.04, p = 0.012; HR = 2.78, p = 0.027), while gender, diabetes, and obesity showed no significant impact. Triglycerides and HDL-c levels notably influenced mortality, with elevated triglycerides and lower HDL-c associated with higher mortality risk (p = 0.032). This study underscores the importance of lipid profiles alongside traditional risk factors in assessing COVID-19 risk and outcomes. It contributes to the understanding of COVID-19 patient management and emphasizes the need for further investigation into the role of dyslipidemia in influencing COVID-19 prognosis, potentially aiding in refined risk stratification and therapeutic strategies.

## Introduction

The COVID-19 pandemic has affected millions worldwide, causing significant morbidity and mortality^1,2^. The SARS-CoV-2 virus, which primarily affects the respiratory system, is responsible for the disease. However, recent studies have shown that the virus also affects lipid metabolism, leading to changes in lipid profile levels^3–15^. Lipids are essential components of lipid rafts and are important in the SARS-CoV-2 infection process^16,17^. Several studies have investigated the relationship between COVID-19 and lipid profile levels, with some suggesting that lipid profile abnormalities are associated with COVID-19 severity and mortality^3,7,9,10,18,19^. A recent systematic review and meta-analysis demostrate that lipid profile abnormalities were significantly related to COVID-19 severity. Low levels of high-density lipoprotein (HDL) and high triglyceride levels, in particular, were significantly associated with COVID-19 severity^10^.

Similarly, a study by Masana et al. found that low HDL and high triglycerides were predictors of COVID-19 severity^19^. In addition to HDL and triglycerides, other lipid profile parameters have also been investigated concerning COVID-19 severity. For example, a study discovered that plasma apolipoprotein concentrations were significantly altered in severe COVID-19 patients^18^. The study included fifty COVID-19 patients admitted to the intensive care unit (ICU) and found that apolipoprotein A1 and apolipoprotein B were significantly decreased in severe COVID-19 patients compared to non-severe patients^18^.

Furthermore, a number of studies have examined the relationship between lipid profile levels and COVID-19 mortality^3,5,12,13^. A study by Mosaad et al. found that plasma lipid profile levels predict disease mortality among COVID-19 patients. The study included 100 COVID-19 patients and found that low HDL and high total cholesterol levels were significantly associated with COVID-19 mortality^3^. The relationship between lipid profile levels and COVID-19 severity and mortality has also been investigated in specific populations. For example, a study by Oliver et al. investigated the Moderna COVID-19 vaccine in the United States and found that the vaccine was effective in preventing COVID-19-related hospitalization and death in individuals with underlying medical conditions, including dyslipidemia. Similarly, a study by Uyaroğlu et al. investigated lipid levels in post-COVID patients and found that hyperlipidemia was common in these patients^20^.

Several mechanisms have been proposed to explain the relationship between COVID-19 and lipid profile levels. One of them is that the virus directly affects lipid metabolism, leading to changes in lipid profile levels. For example, a study by Malik et al. found that COVID-19 was associated with changes in lipid profile levels and acute phase reactants^6^. Another proposed mechanism is that the virus indirectly affects lipid metabolism through inflammation. A study by Jayant found that inflammatory markers were significantly associated with lipid profile abnormalities in COVID-19 patients^21^.

On the other hand, some studies have delved into the relationship between specific lipid profile ratios and COVID-19 severity and mortality. Zhang et al. found that the cholesterol/HDL-c ratio was significantly associated with COVID-19 severity^8^. Furthermore, low levels of HDL and high levels of triglycerides have been associated with COVID-19 severity, whereas low levels of HDL and high levels of total cholesterol have been associated with COVID-19 mortality. However, further research is needed to fully understand the mechanisms underlying the relationship between COVID-19 and lipid profile levels and to develop effective interventions to prevent and treat COVID-19-related dyslipidemia.

Several studies have uncovered the complex relationship between lipid metabolism and COVID-19 outcomes, highlighting the potential predictive value of lipid profiles in classifying patient risk and treatment. As a biomarker, high-density lipoprotein cholesterol (HDL-c) has garnered attention owing to its consistent association with the severity and outcomes of COVID-19 across diverse patient populations. The association between low HDL-c levels and an elevated probability of experiencing severe COVID-19 outcomes was underscored by Wang et al.^22^. Reiterating this, Masana et al. found that having low HDL-c and high triglycerides together could predict the severity of COVID-19^19^. Further substantiating this notion, significant variations in HDL-c levels were observed in individuals afflicted with severe strains of COVID-19, as reported by Begue et al. and Li et al. This finding implies that the virus might exert an influence on lipid metabolism^23,24^.

Moreover, further research has provided further support for the prognostic significance of HDL-c levels. Stewart et al.^25^ associated low HDL-c levels with fatal COVID-19 courses, while Ghoreshi et al.^26^ suggested that the cytokine milieu in COVID-19 could lead to decreased HDL-c levels. Baycan et al.^27^ discussed the long-term mortality implications of HDL-c levels in COVID-19 patients, highlighting its prognostic significance.

Furthermore, the exploration of HDL-c levels in pediatric COVID-19 patients by Mietus-Snyder et al.^28^, and its significant reduction in severe cases as reported by Agouridis et al.^29^ and Erman et al.^30^, extends the relevance of HDL-c as a prognostic factor across age groups. The examination of lipid ratios, such as those undertaken by Rohani-Rasaf et al.^31^ and Alcántara-Alonso et al.^32^, further elucidates the role of lipid profiles in predicting COVID-19 outcomes, with Zhang et al.^24^ emphasizing the TG/HDL-c ratio as a marker of cardiovascular risk and poor prognosis in COVID-19 patients. Regarding this, the body of evidence consistently supports the association between low HDL-c levels and adverse COVID-19 outcomes, underscoring the importance of lipid profiles in the disease's risk stratification and management strategies. Our study builds upon this foundation, aiming to elucidate the role of HDL-c and lipid profiles in COVID-19 within the Mexican population, thereby contributing valuable insights for clinical practice and future research.

The main objective of this study was to investigate the predictive value of HDL-c and lipid profile in influencing the outcomes of COVID-19 patients. The objectives encompass understanding the distribution and impact of these factors across severity and outcome groups, establishing optimal cut-off points for lipid parameters, and investigating the potential of these variables as prognostic markers. Particular attention was paid to potential links between HDL-c levels and mortality risk. Through these objectives, this research aims to provide valuable insights that could inform risk stratification strategies and consequently improve the planning and management of treatment for COVID-19 patients.

## Results

In the current study, 102 COVID-19 patients were involved during the pandemic’s first wave. The cohort was divided by severity into non-critical (65 patients) and critical (37 patients) conditions and by outcome into survivors (64 patients) and non-survivors (38 patients). According to Pearson's Chi-squared test, there was no statistically significant difference in gender distribution between these divisions, with approximately 36% female and 64% male (Table 1). Age was identified as a significant prognostic factor for both severity and mortality, with median ages of 61 and 63 years for critical and non-surviving patients, respectively, compared to 54 and 53 years for their non-critical and surviving counterparts (p-value for severity = 0.007; p-value for mortality < 0.001). Hypertension was another variable significantly correlated with both severity and mortality, with 76% of critical cases and 79% of non-survivors had hypertension (p-value for severity = 0.006; p-value for mortality < 0.001). Severity itself was found to be a strong predictor of mortality, with 76% of critical cases resulting in death compared to only 12% in non-critical cases (p-value < 0.001). Notably, variables such as gender, diabetes, and obesity were not statistically significant in predicting either severity or mortality. Regarding comorbidities, a substantial proportion of the cohort had obesity and was uniformly distributed, between severity and mortality (Table 1).

**Table 1:** Comparative analysis of demographic, comorbidity and laboratory parameters in COVID-19 patients based on severity and survival status.

Variables	COVID-19 Severity				COVID-19 Mortality		
	Overall n = 102^a^	Non critical n = 65^a^	Critical n = 37^a^	p-value^b^	Survivor n = 64^a^	Non survivor n = 38^a^	p-value^b^
Sex				0.9			> 0.9
Female	37 (36%)	24 (37%)	13 (35%)		23 (36%)	14 (37%)	
Male	65 (64%)	41 (63%)	24 (65%)		41 (64%)	24 (63%)	
Age	57 (46, 64)	54 (42, 62)	61 (52, 68)	0.007	53 (41, 62)	63 (52, 71)	< 0.001
Severity							< 0.001
Non-critical					56 (88%)	9 (24%)	
Critical					8 (12%)	29 (76%)	
Diabetes				0.2			0.3
Not	66 (65%)	45 (69%)	21 (57%)		44 (69%)	22 (58%)	
Yes	36 (35%)	20 (31%)	16 (43%)		20 (31%)	16 (42%)	
Hypertension				0.006			< 0.001
No	43 (42%)	34 (52%)	9 (24%)		35 (55%)	8 (21%)	
Yes	59 (58%)	31 (48%)	28 (76%)		29 (45%)	30 (79%)	
Obesity				0.5			0.6
Not	86 (84%)	56 (86%)	30 (81%)		55 (86%)	31 (82%)	
Yes	16 (16%)	9 (14%)	7 (19%)		9 (14%)	7 (18%)	
Mortality				< 0.001			
Non-survivor	38 (37%)	9 (14%)	29 (78%)				
Survivor	64 (63%)	56 (86%)	8 (22%)				

^a^n (%); Median (IQR).

^b^Pearson's Chi-squared test; Wilcoxon rank sum test.

**Figure 1:**
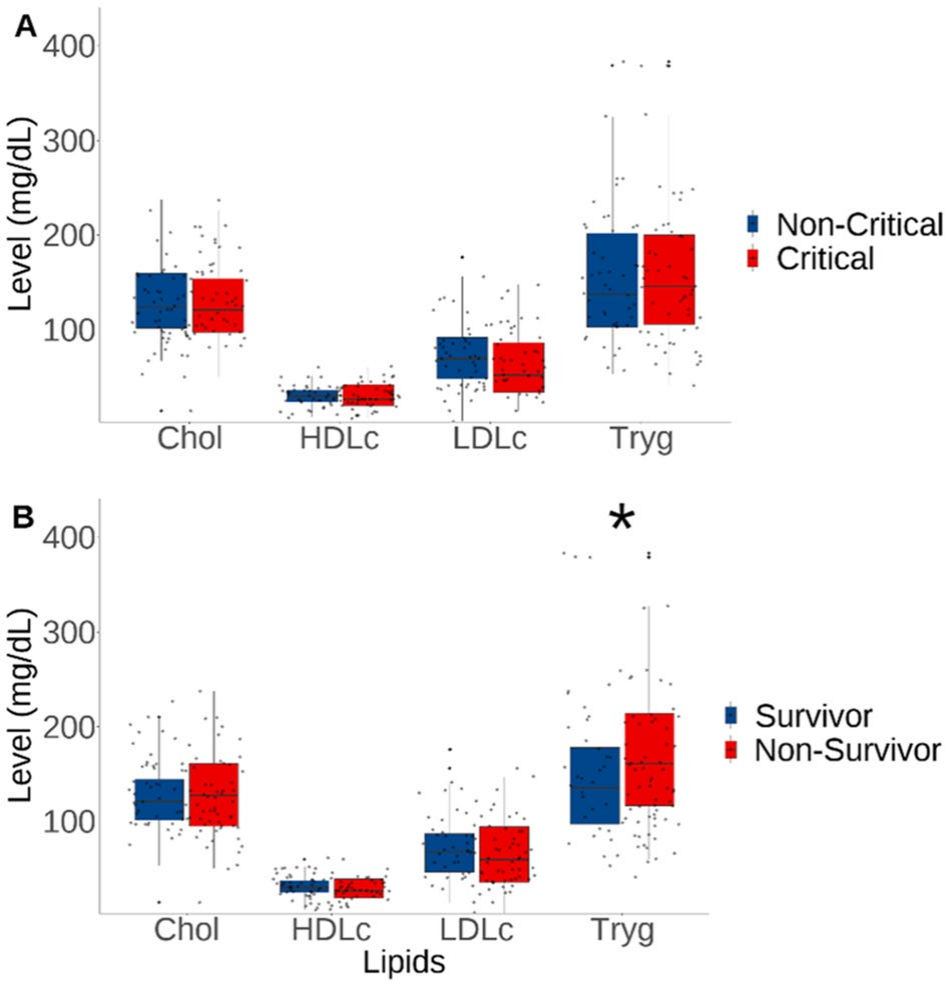
Comparative analysis of lipid profile among COVID-19 severity (**A**) and survival outcome (**B**). Asterisks indicate statistical significance between groups (*p < 0.05).

Subsequently, the lipid profile of COVID-19 patients between severity groups (non-critical vs. critical, Fig. 1A) and outcome groups (survivor vs. non-survivor, Fig. 1B) were compared. No significant differences in cholesterol (Chol) levels across severity or outcome groups were found. The median Chol levels were comparable across all groups, with non-critical patients exhibiting slightly higher levels (124 mg/dL) than critical patients (121 mg/dL) (Fig. 1A). A similar pattern emerged when comparing survivors (121 mg/dL) and non-survivors (128 mg/dL) (Fig. 1B). The High-Density Lipoprotein cholesterol (HDL-c) analysis revealed no significant differences among severity and outcome groups. The values were closely matched in all groups, with non-critical patients and survivors marginally higher (30 mg/dL) than their counterparts. Analyzing LDL cholesterol (LDL-c) showed non-significant, albeit slightly lower median values in the critical (52 mg/dL) compared to non-critical patients (69 mg/dL). The mortality groups showed no significant difference in LDL cholesterol.

Lastly, for triglycerides (Tryg), we observed a significant difference in the outcome groups. Non-survivors showed noticeably higher median levels (161 mg/dL) than survivors (136 mg/dL), indicating a possible association of elevated Tryg with a higher mortality risk in COVID-19 patients (p = 0.032). There was no marked disparity in triglyceride levels between non-critical and critical groups.

**Figure 2:**
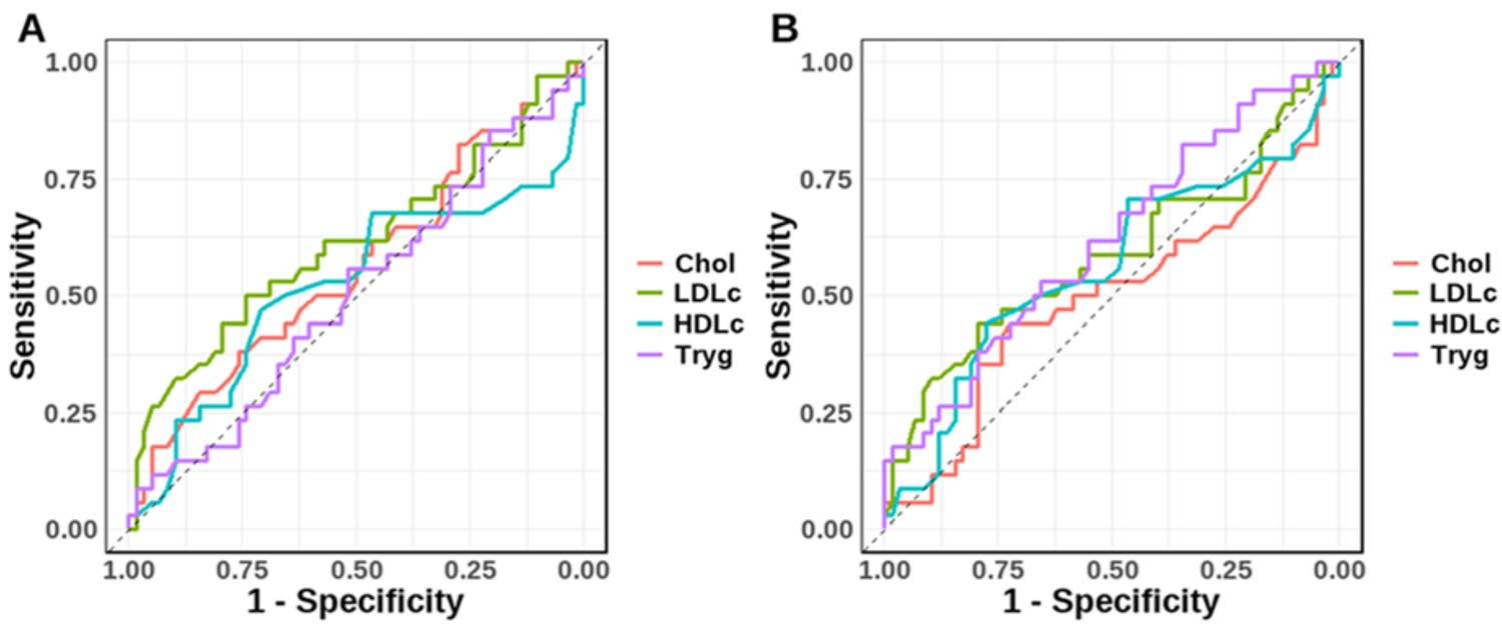
The Receiver Operating Characteristic (ROC) curves for each lipid profile variable. Cholesterol (Chol), Low-Density Lipoprotein (LDL-c), High-Density Lipoprotein (HDL-c), and Triglycerides (Tryg) present a comparison between their ability to predict two different conditions: severity (**A**) and patient survival outcome (**B**).

The evaluation of the Area Under the Receiver Operating Characteristic Curve (AUC-ROC) further substantiated the discriminatory power of these variables. In the severity context, the AUC values for Chol, LDL-c, HDL-c, and Tryg were 0.86, 0.79, 0.81, and 0.88, respectively (Fig. 2A). Likewise, in the survival outcome context, the AUC values were 0.87, 0.82, 0.82, and 0.89, respectively (Fig. 2B).

In the current study, we performed univariate and multivariate logistic regression analyses to examine the relationship between several dichotomous lipid variables—Chol, LDL-c, HDL-c, Tryg—and COVID-19 severity and mortality, while adjusting for potential confounders such as age, sex, Diabetes, Hypertension, and obesity. Logistic regression analysis was applied to determine optimal cut-off points for each lipid parameter. This procedure revealed moderate discriminatory ability for all lipid profile components.

Before the analysis, optimal cut-off points for each lipid variable were established based on Youden's index J: 99.5 mg/dL for Chol, 48.5 mg/dL for LDL-c, 25.5 mg/dL for HDL-c, and 139.5 mg/dL for Tryg in the severity context. Similarly, for the survival context, the cut-off points were 138.5 mg/dL, 44.5 mg/dL, 24.5 mg/dL, and 156.5 mg/dL, respectively. The subsequent dichotomization of these variables was carried out using these cut-off values (Table 2). The logistic regression analyses yielded intriguing findings as each lipid variable was significantly associated with the mortality outcome, except for Tryg. The multivariate logistic regression model revealed that, after adjusting for age, sex, Diabetes, Hypertension, and obesity, the lipid variables had substantial predictive value to severity and mortality outcome (Table 2). To validate the results, we performed variance inflation factor (VIF) analyses, confirming the minimal multicollinearity among predictors in both models (VIF values ranged from 1.003763 to 1.027953), confirming the absence of significant collinearity issues that could impact the interpretation of our results (Supplemental Material). These findings validate the statistical integrity of our models and support the reliability of our conclusions regarding the predictive value of HDL-c and other lipid profiles in COVID-19 outcomes. We visualized the discriminative ability of our logistic regression models through ROC curves. The AUC for the Clinic Model was found to be 0.5758, indicating a limited yet better-than-random ability to distinguish between the levels of disease severity. The AUC for the Outcome Model was slightly higher at 0.6375, suggesting a moderate predictive ability (Supplemental Material). Additionally, to enhance the reliability of our logistic regression model, we performed a k-fold cross-validation with 100 iterations, which further substantiated its stability across diverse subsets of data. The cross-validation results showed an average ROC of 0.5753, which was very close to the AUC found in the first ROC analysis (Supplemental Material). These results shows that the model's performance is consistent. The sensitivity of the model was notably high at 0.9855, suggesting an excellent ability to identify true positive cases. However, the specificity was markedly low at approximately 0.0127, indicating a limitation in accurately identifying true negative cases, which could potentially lead to overestimations of risk in lower-risk populations.

**Table 2:** Univariate and multivariate cox proportional hazards regression analysis for COVID-19 patient survival based on demographics, lipid profiles, and comorbidities.

Variables	COVID-19 severity			COVID-19 mortality		
	Log (OR)^a^	95% CI^a^	p-value	Log (OR)^a^	95% CI^a^	p-value
Age	0.08	0.03, 0.13	0.003	0.09	0.03, 0.15	0.003
Sex						0.5
Female	–	–		–	–	
Male	0.20	− 0.99, 1.4	0.7	0.50	− 0.80, 1.9	
Chol ≥ 99.5	0.91	− 0.79, 2.8	0.3			
LDL-c ≤ 48.5	2.2	0.53, 4.2	0.015			
HDL-c ≤ 25.5	1.1	− 0.07, 2.4	0.073			
Tryg ≥ 139.5	1.0	− 0.09, 2.3	0.082			
Diabetes						0.5
No	–	–		–	–	
Yes	0.08	− 1.0, 1.2	0.9	− 0.45	− 1.8, 0.80	
Hypertension						0.014
No	–	–		–	–	
Yes	1.6	0.33, 3.1	0.020	1.8	0.43, 3.3	
Obesity						0.6
No	–	–		–	–	
Yes	0.69	− 0.81, 2.2	0.4	0.51	− 1.2, 2.3	
Chol ≥ 138.5				3.1	1.3, 5.3	0.002
LDL-c ≤ 44.5				2.6	0.97, 4.5	0.004
HDL-c ≤ 24.5				2.6	1.0, 4.5	0.003
Tryg ≥ 156.5				0.65	− 0.72, 2.1	0.4

^a^*OR* Odds ratio, *CI* confidence interval.

In light of the aforementioned findings, an alternative statistical method such as Cox regression for time-to-event data was included, which might better capture the dynamics of our study's endpoints. This approach is particularly relevant given the temporal nature of clinical outcomes in our dataset. Regarding the mortality outcome context, the binary variables created using the cut-off values demonstrated significant associations with COVID-19 mortality. The multivariate logistic regression model, adjusted for the same potential confounders, indicated that the lipid variables, particularly Chol ≥ 138.5 mg/dL and LDL-c ≤ 44.5 mg/dL, predicted the mortality outcome (Table 2). In both outcome contexts, Tryg ≥ 139.5/Tryg ≥ 156.5 displayed the highest AUC, indicating its superior performance in distinguishing between patients with different COVID-19 severity and survival outcomes. Conversely, LDL-c ≤ 48.5/LDL-c ≤ 44.5 exhibited the lowest AUC values, suggesting its comparative inferiority (Fig. 2).

Moreover, survival analyses were performed for each lipid variable based on the established cut-off points. Kaplan–Meier survival curves were plotted, clearly illustrating the survival probabilities over time for the two groups in each lipid variable, dichotomized based on the corresponding cut-off point.

**Figure 3:**
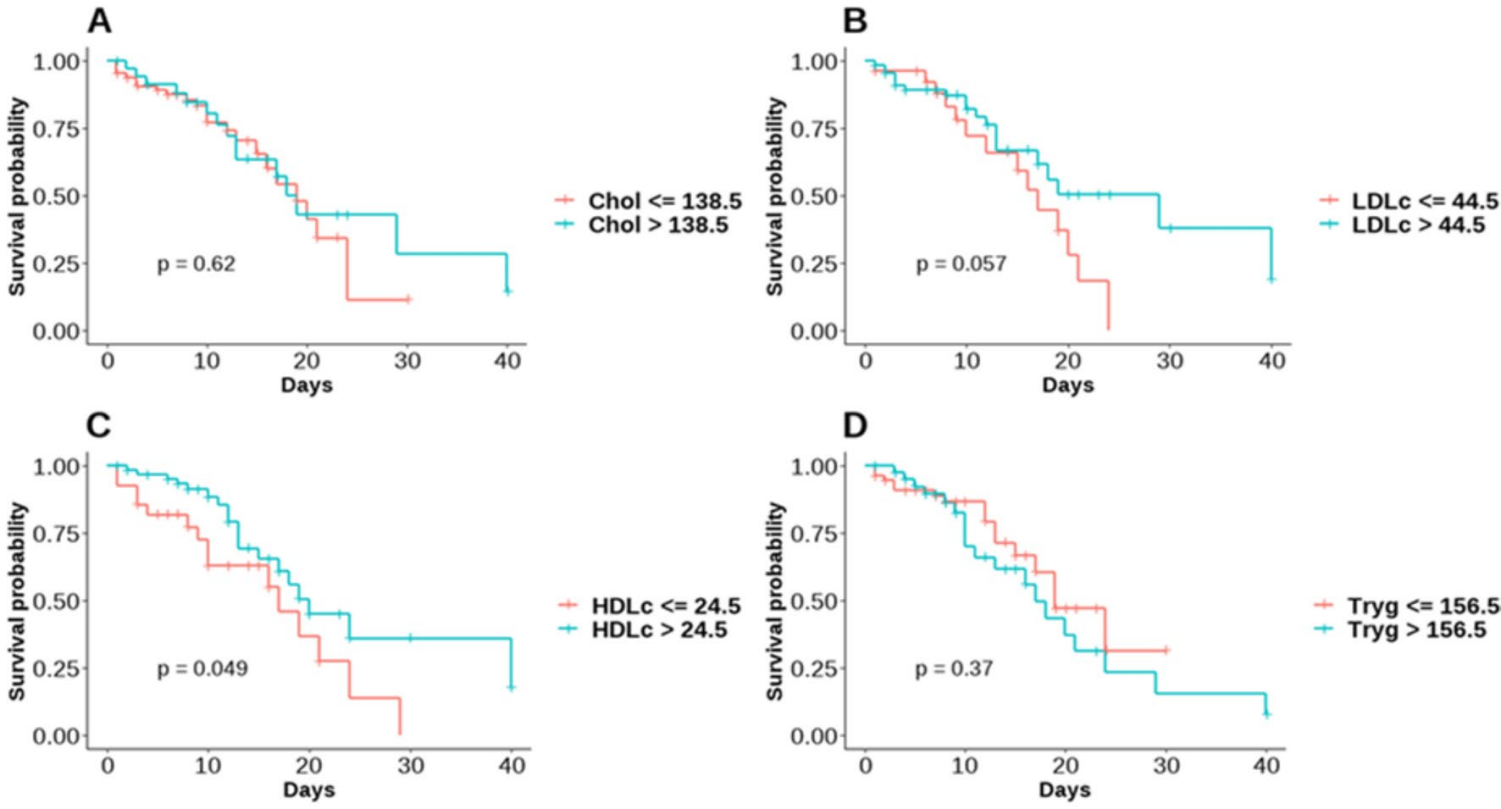
Kaplan–Meier Survival Curves for dichotomized lipid variables [(**A**) Cholesterol (Chol), (**B**) Low-Density Lipoprotein (LDL-c), (**C**) High-Density Lipoprotein (HDL-c), and (**D**) Triglycerides (Tryg)] in the context of COVID-19 survival outcome.

Figure 3A–D shows the survival probability over time, as depicted by Kaplan–Meier survival curves, exhibiting distinct trends between groups separated by lipid level-determined cut-off points. As demonstrated, differences in cholesterol and triglyceride levels did not appear to affect survival probabilities (p > 0.05) (Fig. 3 A and D). Both groups, defined by these parameters, exhibited comparable survival probabilities, indicating that cholesterol and triglyceride levels have little effect on patient survival. In stark contrast, survival probabilities based on LDL-c levels displayed intriguing tendencies. When the cohort was divided into "LDL-c ≤ 44.5" and "LDL-c > 44.5" groups, distinct survival probabilities emerged (Fig. 3B). As indicated by the p-value of 0.05 for the log-rank test, which is close to statistical significance, the group with LDL-c > 44.5 had marginally better outcomes (Fig. 3B). On the other hand, the simultaneous analysis of survival probabilities based on HDL-c levels revealed significant insight. When divided into groups with HDL-c ≤ 24.5 and HDL-c > 24.5, survival curves diverged dramatically (Fig. 3C). A p-value of 0.049 for the log-rank test indicated a statistically significant difference between these groups. The above result suggests that COVID-19 patients with higher HDL-c levels may have a greater chance of survival.

Univariate and multivariate Cox proportional hazards regression models were subsequently employed to evaluate these potential predictors. The Cox proportional hazards regression models, both univariate and multivariate, were used to investigate these potential predictors further. In the univariate analysis, age (HR 1.04, 95% CI 1.02–1.06, p = 0.001) and Hypertension (HR 3.12, 95% CI 1.37–7.12, p = 0.007) emerged as significant predictors of survival. When adjusting for other factors in the multivariate model, the impact of age (HR 1.04, 95% CI 1.01–1.08, p = 0.012) and Hypertension (HR 2.78, 95% CI 1.12–6.91, p = 0.027) continued to be statistically significant (Table 3). Other factors, such as sex, diabetes, and obesity, did not exhibit significant associations with survival outcomes in the univariate or multivariate model (Table 3). Although not significant predictors in the multivariate model, the lipid variables warrant further investigation due to their clinical relevance. The determined cut-off points did not yield significant results in the present study but could be valuable in the context of other research frameworks or patient populations.

**Table 3:** Hazard ratios (HR) and 95% confidence intervals (CI) from univariate and multivariate cox proportional hazards regression models for COVID-19 patient survival outcomes based on demographic characteristics, lipid profile parameters, and comorbidities.

Variables	Univariate model				Multivariate model		
	N	HR^a^	95% CI^a^	p-value	HR^a^	95% CI^a^	p-value
Age	102	1.04	1.02, 1.06	0.001	1.04	1.01, 1.08	0.012
Sex	102						
Female		–	–		–	–	
Male		0.92	0.46, 1.82	0.8	1.23	0.58, 2.61	0.6
Chol ≥ 138.5	102	0.84	0.42, 1.65	0.6	1.62	0.45, 5.79	0.5
LDL-c ≥ 44.5	94	0.52	0.26, 1.03	0.059	0.46	0.15, 1.46	0.2
HDL-c ≥ 24.5	93	0.51	0.26, 1.01	0.054	0.52	0.22, 1.25	0.14
Tryg ≥ 156.5	98	1.35	0.69, 2.67	0.4	1.49	0.58, 3.82	0.4
Diabetes	102						
Not		–	–		–	–	
Yes		1.09	0.56, 2.11	0.8	0.93	0.43, 2.04	0.9
Hypertension	102						
Not		–	–		–	–	
Yes		3.12	1.37, 7.12	0.007	2.78	1.12, 6.91	0.027
Obesity	102						
Not		–	–		–	–	
Yes		0.89	0.38, 2.06	0.8	1.83	0.63, 5.27	0.3

^a^*HR* Hazard ratio, *CI* confidence interval.

## Discussion

This research provide a comprehensive examination of the impact of clinical, demographic, and lipid-related factors on the severity and mortality of patients with COVID-19. The findings presented in our study indicate that there are no statistically significant differences between critical and non-critical cases when considering gender. Nevertheless, it is worth noting that there was a discernible pattern linked to age, as older people were overrepresented among patients who experienced severe outcomes or did not survive. These data provide support for the widely accepted scientific consensus that age is a significant risk factor for negative outcomes related to COVID-19, such as hospitalization, admission to critical care units, and death^33^.

The substantial proportion of the cohort with diabetes and hypertension in this study is consistent with previous research indicating that these comorbidities are associated with worse outcomes in COVID-19^34^. These conditions are known to exacerbate the inflammatory response and contribute to endothelial dysfunction, which may enhance the severity of SARS-CoV-2 infection. This suggest that COVID-19 patients with comorbidities like diabetes and hypertension may be at higher risk of disease severity and mortality^35^. Therefore, it is significant to identify and manage these comorbidities in COVID-19 patients to improve their outcomes.

However, variables including sex, diabetes, and obesity, were not significantly associated with survival outcomes. Previous studies have identified these as potential risk factors for severe COVID-19 outcomes^36,37^, which is unexpected. The absence of an association in the present study may be attributable to the small sample size, necessitating additional research in larger cohorts.

When comparing the lipid profiles of COVID-19 patients between severity and survival outcome groups, no significant differences in cholesterol levels across severity or outcome groups in COVID-19 patients. The median cholesterol levels were comparable across all groups, with non-critical patients exhibiting slightly higher levels than critical patients. Similarly, the study found no significant differences in high-density lipoprotein cholesterol (HDL-c) levels among severity and outcome groups. The values were closely matched in all groups, with non-critical patients and survivors marginally higher than their counterparts. The study found slightly lower median values of low-density lipoprotein cholesterol (LDL-c) in critical compared to non-critical patients, but this difference was insignificant. This finding is consistent with a study by Zhang et al. that found that LDL-c levels were lower in COVID-19 patients with severe disease than those with mild disease^8^.

Triglyceride levels showed a statistically significant difference between survival outcome groups, with non-survivors exhibiting higher median levels. This suggests a potential association between elevated triglycerides, reduced HDL-c levels, and increased mortality risk in COVID-19 patients. According to additional studies, high triglyceride levels and low HDL-c levels are predictors of COVID-19 severity and mortality^6,10,19,38^. Nevertheless, the relationship between lipid profiles and COVID-19 outcomes is not yet fully understood, and additional research is required to identify the precise mechanisms underlying this association. According to the lipid profile changes, elevated triglycerides are associated with COVID-19 severity and mortality; this finding is consistent with a recent meta-analysis that linked elevated triglycerides to COVID-19 severity and mortality^10^.

Similarly, Masana et al.^19^ identified low HDL cholesterol and elevated triglycerides as predictors of COVID-19 severity. However, the conclusions drawn from previous studies regarding the alterations in serum lipids in COVID-19 patients are inconsistent. For instance, Liu et al. found that dyslipidemia was not associated with COVID-19 severity or mortality^39^. Different study designs, patient populations, and lipid profile measurements may account for these discrepancies.

The present study also evaluated the ability of lipid profile variables to predict COVID-19 severity and mortality using AUC-ROC curves. The study found that all lipid profile components had moderate discriminatory ability, with AUC values ranging from 0.79 to 0.89. The study also established optimal cut-off points for each lipid variable based on the Youden's index J and found that each lipid variable, except for Tryg, showed a significant association with mortality outcome in logistic regression analysis. The multivariate logistic regression model revealed that the lipid variables had substantial predictive value for severity and mortality outcome, even after adjusting for potential confounders such as age, sex, diabetes, hypertension, and obesity.

These findings are consistent with previous research showing that lipid profile alterations are associated with COVID-19 severity and mortality^40^. Moreover, the present study's findings suggest that lipid profile variables may be useful in predicting COVID-19 outcomes and could be used as a readily available biological marker to predict the severity and mortality of COVID-19 infection^41^. However, it is essential to note that lipid profile variables are just one of many factors that may influence COVID-19 outcomes. Other factors, such as treatment adherence on chronic diseases^42^, albumin levels^43^, Leukocyte glucose index^44^, and neutrophil–lymphocyte ratio^41^, have also been identified as predictors of COVID-19 outcomes.

Other research indicates that comorbidities may moderate the relationship between lipid profile and COVID-19 outcomes. For instance, Kumari et al.^45^ found that lipid profiles may be a potential marker for determining the disease prognosis for COVID-19 patients, but they did not examine the impact of comorbidities. The precise mechanisms coupling these comorbidities to worse outcomes are not fully understood, but they could involve a combination of factors, such as chronic inflammation, impaired immune function, and increased susceptibility to viral entry and replication.

In addition, the findings of the presented study may have substantial implications for the treatment of COVID-19 patients. Age and hypertension were identified as significant survival predictors in COVID-19 patients, particularly in the context of early identification and management of comorbidities. Findings regarding triglycerides suggest that surveillance and treatment of lipid profile abnormalities may be necessary for enhancing COVID-19 outcomes. In addition, further research is necessary to thoroughly comprehend the mechanisms underlying the association between COVID-19 and lipid profile levels and to develop effective interventions for preventing and treating dyslipidemia caused by COVID-19. Furthermore, this study emphasizes the significance of considering comorbidities such as diabetes and hypertension when assessing the severity and mortality of COVID-19. Age and hypertension were significant survival predictors in both univariate and multivariate Cox proportional hazards regression models. Additionally, the survival analysis for HDL-c levels also revealed interesting findings.

The present study found that COVID-19 patients with higher HDL-c levels may have a greater chance of survival. This finding aligns with previous research suggesting that HDL-c, known for its anti-inflammatory and antioxidant properties, may have a protective role in COVID-19. In this sense, some studies evaluated the HDL-c anti-inflammatory and antioxidant activity, proving that the Serum amyloid A (SAA), Apolipoprotein A-1, Alpha-1 antitrypsin, and paraoxonase 1 (PON-1) (HDL-c associated proteins) are altered in SARS-CoV-2 infection which could be related to decreased functionality of HDL-c in COVID-19 severity^23,46^.

Moreover, evidence suggests that cholesterol-rich lipid rafts and receptors, such as HDL scavenger receptor B type 1 (SR-B1), which regulate lipid entry into cells can enhance SARS-CoV-2 entry^17^. HDL-c mobilizes the cholesterol of cholesterol-rich lipid rafts for traffic and re-localizes SARS-CoV-2 receptors, which promote the viral entry into cells^17^. Because HDL-c has immunomodulatory effects, it could be hypothesized that low HDL-c levels during infection are associated with the regulation of immune cells in COVID-19 severity^47,48^. A study evaluating lymphocytes, macrophage activation, dendritic cells, inflammatory mediators, cytokines, and their correlation with HDL-c levels during SARS-CoV-2 infection is required to test this hypothesis.

Stadler et al. demonstrated that the cholesterol efflux capacity is associated with mortality, ApoA-I protein, HDL-ApoA-I protein, HDL-c, total ApoA-II protein, HDL-free cholesterol, and HDL phospholipids in patients with COVID-19^49^. Another study demonstrating the importance of HDL-c during SARS-CoV-2 infection showed that a ratio of triglyceride to HDL-c was related to the risk of severe COVID-19^50^.

Changes in lipid profile levels could have clinical applicability in providing timely treatment for patients with COVID-19. In this regard, a study of lipid profile trajectories during the two years before COVID-19 testing revealed that higher antecedent HDL-c levels were associated with a lower SARS-CoV-2 infection risk. These levels, however, declined during viral infection^51^. On the other hand, Jin et al. reported that the patients infected during the first wave of COVID-19 with high levels of low-density lipoprotein cholesterol (LDL-c), triglyceride, and total cholesterol before infection and on admission had a poor progression of COVID-19^7^. Moreover, HDL-c, LDL-c, total cholesterol, and triglyceride were significantly lower in the patients with COVID-19 during the first wave of COVID-19, demonstrating that lipid profile predicts the severity of SARS-CoV-2 infection^52^. Al-Zadjali et al. demonstrated that low HDL-c levels are associated with increase long-term COVID-19 severity in unvaccinated patients infected with SARS-CoV-2 after the first wave of COVID-19^53^. Regarding vaccination, a study focused on analyzing the lipid profile before and after the two doses of the COVID-19 vaccination in patients without exposure to SARS-CoV-2 infection revealed that triglyceride levels were significantly decreased and cholesterol, HDL-c, and LDL-c levels were significantly increased in patients who received the mRNA-1273 (Moderna) vaccine^54^. Individuals vaccinated with the BNT162b2 (Pfizer-BioNTech) vaccine had a significant increase in HDL-c, while patients vaccinated with ChAdOx1 nCov-19 (Oxford-AstraZeneca) had no change in lipid profile after follow-up^54^. Szczerbiński et al. reported an absence of statistically significant correlation between the total cholesterol, HDL-c, and LDL-c levels and anti-SARS-CoV-2 S antibodies concentration at the end-of-observation-19 weeks in patients vaccinated with the second dose of BNT162b2 mRNA COVID-19 vaccine^55^. On the other hand, in the patients who received the COVID-19 vaccine and were diagnosed with SARS-CoV-2 infection after the first wave of COVID-19, the total cholesterol, HDL-c, and LDL-c were significantly lower in non-survivors and these values were associated with the mortality risk^56^. Furthermore, patients with low levels of LDL-c, total cholesterol, and anti-SARS-CoV-2 antibodies had the highest mortality rates^56^. Thus, lipid profile, emerging SARS-CoV-2 variants, and the immune response in COVID-19 vaccine recipients (neutralizing antibodies against SARS-CoV-2) could be strongly related with COVID-19 mortality through a pathophysiological mechanism where the statin therapy would improve the chances of survival^57^.

Despite the emergence of new SARS-CoV-2 variants, our findings remain clinically relevant, offering insights into the prognostic value of lipid profiles in COVID-19 patients. These insights are pivotal for risk stratification and the development of management strategies that are adaptable to the evolving pandemic landscape. Furthermore, this study elucidates the critical associations between lipid profile abnormalities and COVID-19 severity and mortality, thereby contributing significantly to existing literature. However, the study is not without limitations. The retrospective design, small sample size, and absence of control groups introduce potential biases and limit the generalizability of our findings. Additionally, uncontrolled confounding variables and population heterogeneity further constrain the study's applicability. Despite these constraints, this research makes a significant contribution to the field. Future investigations should adopt a prospective methodology, incorporate larger and more diverse cohorts, and include control groups. Employing advanced statistical techniques to control confounders and conducting mechanistic studies will refine our understanding and facilitate targeted interventions, thereby enhancing the study's global scientific impact.

Our findings suggest that patients with comorbidities should be classified with caution based on their lipid profile values when assessing the prognosis of COVID-19 patients. In addition, the relationship between lipid profile, disease outcome, and comorbidities must be understood to guide adequate risk stratification and treatment planning for COVID-19 patients. Similarly, the underlying mechanisms and potential interventions for dyslipidemia in COVID-19 patients require additional research. Despite its moderate AUC, the logistic regression model suggests a limited yet non-negligible ability to discriminate between patient outcomes based on lipid profiles. While the model exhibits high sensitivity, its specificity is notably low, indicating a propensity for false positive predictions, which could limit its clinical utility. In contrast, the Cox model's incorporation of time-to-event data provides a more granular analysis of risk factors, reinforcing the prognostic significance of age and hypertension. These results are consistent with existing literature that highlights the exacerbation of COVID-19 severity by underlying health conditions. Our findings corroborate previous studies that have identified age and hypertension as critical determinants of COVID-19 prognosis. However, the unique contribution of this study lies in its analytical approach, combining logistic regression with Cox proportional hazards modeling to enhance the depth of prognostic assessments. This study's primary limitations stem from its reliance on available clinical data, which may not capture all potential confounders. Future research should explore the inclusion of additional variables, such as genetic markers and patient lifestyle factors, which could further refine the predictive models presented herein. However, this study advances knowledge by shedding light on the prognostic significance of the lipid profile of COVID-19 infection. It also highlights the significance of considering lipid metabolism in treating and staging the disease.

When understanding the usefulness of lipid profiles in the prognosis of COVID-19, the classification of patients based on clinical guidelines or other clinical parameters should be considered. Our study classified the patients according to the “Diagnosis and Treatment Protocol for Novel Coronavirus Pneumonia,” issued by the Chinese Centers for Disease Control and Prevention. Other studies that validated a clinical risk score or analyzed intermediates of cholesterol biosynthesis and their association with the clinical outcomes of SARS-CoV-2 infection classified the severity of COVID-19 based on the American Thoracic Society guidelines and the National Institutes of Health recommendations, respectively^58,59^. Hence, these and other classifications should be considered when comparing study results to avoid errors in interpreting severity and mortality predictors for COVID-19.

A comprehensive understanding of the effect of lipid profiles on COVID-19 outcomes may lead to improved clinical decision-making and patient care. In conclusion, this study advances our understanding of the factors influencing COVID-19 outcomes, emphasizing the role of systemic health issues such as hypertension. The dual analytical approach utilized herein not only highlights significant predictors but also underscores the complexity of prognostic modeling in infectious diseases.

## Methods

### Study design and participants

A total of 102 COVID-19 patients were enrolled in the study. The patients were admitted to a designated COVID-19 treatment center in Hospital General de Culiacan, Sinaloa, “Bernardo J. Gastelum,” during the study period. The COVID-19 patients who met any of the following criteria were excluded from the study: (1) pregnant patients; (2) patients ages ≤ 18 years; (3) patients having severe medical conditions, including chronic renal dysfunction, malignant tumor, acquired immune deficiency syndrome, and liver cirrhosis; and (4) patients with essential information deficits. Given the retrospective nature of the research, the Research Ethical Committee of the Hospital approved the wavier for informed consent.

### Classification of COVID-19 severity

Disease severity was defined according to “Diagnosis and Treatment Protocol for Novel Coronavirus Pneumonia”, issued by the Chinese Centers for Disease Control and Prevention. Patients with a confirmed diagnosis of COVID-19 were classified into four types: (1) mild, patients with slight clinical symptoms and no imaging finding of pneumonia; (2) moderate, patients with fever and respiratory symptoms, and signs of pneumonia on radiologic assessment; (3) severe, patients met any of the following criteria [(a) shortness of breath, RR ≥ 30 times/min; (b) oxygen saturation ≤ 93% at rest; and (c) partial pressure of oxygen (PaO_2_)/fraction of inspired oxygen (PaO_2_/FiO_2_ ≤ 300 mmHg); (d) pulmonary imaging showing the significant progression of lesion > 50% within 24–48 h]; and (4) critical, patients showing any of the following conditions (respiratory failure requires mechanical ventilation, shock, combined with other organ failure requires intensive care and treatment. For further analysis in this study, the patients were grouped as “non-severe” (classified as mild or moderate type) and “severe” (classified as severe or critical type) according to other reports where some factors were used to predict severe COVID-19.

### Follow-up and outcomes

Patients were followed-up for 40-day all-cause mortality. The group of non-survivors were identified by review of electronic medical records. Follow-up adjudication was conducted by investigators who were blinded to hematological parameters measurements.

### Laboratory analysis

COVID-19 was confirmed with a positive result on the RT-qPCR. The clinical sample collection, processing, and laboratory testing were based on WHO guidelines. Viral RNA purification was performed by the QIAamp Viral RNA Mini Kit (QIAGEN) according to the manufacturer’s instructions. The RT-qPCR was performed with the nucleic acid testing kit (Daan, Guangzhou, China) to amplify regions of the open reading frame 1 ab (ORF1ab) and nucleocapsid protein of SARS-CoV-2.

Blood samples were obtained upon each patient´s admission in the Emergency Department and analyzed to determine the levels of hematological parameters. All samples were processed in the same manner.

### Lipid profile determination

Blood samples were collected from all the patients upon admission. The lipid profile, including cholesterol, high-density lipoprotein cholesterol (HDL-c), low-density lipoprotein cholesterol (LDL-c), and triglycerides, were determined based on an immunoanalytical method using a Roche/Hitachi cobas c501 automatic analyzer. The lipid profile was compared between the severity groups (non-critical vs. critical) and the outcome groups (survivor vs. non-survivor).

### Data collection

All data from the patients meeting the inclusion criteria were abstracted from the electronic medical records. Clinical parameters included age, sex, comorbidities, signs and symptoms, laboratory results, and vital signs were collected on admission. Recognizing the potential impact of comorbidities as confounders, our analysis was enriched with a detailed examination of conditions such as diabetes and hypertension. Although the retrospective design limits our ability to fully account for all confounders, such as the days of patient symptoms, previous history of dyslipidemia, use of antihyperglycemic and antidyslipidemic medications, medication uptake, this comprehensive approach strengthens our findings and provides a more nuanced understanding of the relationship between lipid profiles and COVID-19 severity.

### Statistical analysis

Descriptive statistics were used to summarize the demographic and clinical characteristics of the patients. Pearson's Chi-squared test was used to compare categorical variables, while the Wilcoxon rank-sum test was used for continuous variables. Logistic regression analysis was used to determine the optimal cut-off points for each lipid parameter. Receiver Operating Characteristic (ROC) curves were constructed using these thresholds. Survival probability over time was visualized using Kaplan–Meier survival curves, and the Cox proportional hazards regression models was used to investigate potential predictors. To ensure the integrity of our findings, we conducted rigorous statistical analyses models, including assessments for colinearity among independent variables and adjustments for multiple comparisons using the Bonferroni correction method.  Additionally, k-fold cross validation were performed. All statistical analyses were performed using the statistical R programming language version 4.2.2 and related packages in RStudio last version software, and a p-value of less than 0.05 was considered statistically significant.

### Ethics approval

This study was approved by the Ethics Committee of Hospital General de Culiacan, Sinaloa, “Bernardo J. Gastelum,” including the exemption of the requirement for informed consent. The study was compliant with the Declaration of Helsinki. We certify that all protocols and methods were carried out under relevant guidelines and regulations. Due to Mexican laws, the research team cannot share the complete database used for the current paper. Since the number of patients included in this study was limited, the data could contain potentially identifying or sensitive patient information. Nevertheless, other researchers who meet the criteria may request access to the minimal data set underlying the results under request at the Ethics Committee.

### Supplementary Information

Supplementary Information.

## Data Availability

The datasets generated and analyzed during the study are available from the corresponding author upon reasonable request.
