# Supplementary material for: Association between lipid profile and clinical outcomes in COVID-19 patients
Source: Sci Rep. 2024 May 27;14:12139. doi: 10.1038/s41598-024-62899-y (PMC11130121; doi:10.1038/s41598-024-62899-y)
Supplement: Supplementary file 1 — Supplementary Information. [file 41598_2024_62899_MOESM1_ESM.docx]

**Association Between Lipid Profile and Clinical Outcomes in COVID-19 Patients**

Luis Antonio Ochoa-Ramírez^1^, Alberto Kousuke De la Herrán Arita^2^, Jorge Guillermo Sanchez-Zazueta^3^, Efrén Ríos-Burgueño^4^, Joel Murillo-Llanes^5^, Luis Adrián De Jesús-González^6^, Carlos Noe Farfan-Morales^7^, Carlos Daniel Cordero-Rivera^8^, Rosa María del Ángel^8^, Alejandra Romero-Utrilla^9^, Josué Camberos-Barraza^2^, Marco Antonio Valdez-Flores^2^, Alejandro Camacho-Zamora^2^, José Candelario Batiz-Beltrán^2^, Carla Angulo-Rojo^2^, Alma Marlene Guadrón-Llanos^2^, Verónica Judith Picos-Cárdenas^2^, Claudia Desiree Norzagaray-Valenzuela^3^, Ángel Radamés Rábago-Monzón^2^, Jesús Salvador Velarde-Félix^1,3^, José Manuel Reyes-Ruiz^10,11^*, and Juan Fidel Osuna-Ramos^2,^*

1. Hospital General de Culiacán “Bernardo J. Gastelum”, Servicios de Salud de Sinaloa. Culiacán, Sinaloa, México.
2. Facultad de Medicina, Universidad Autónoma de Sinaloa, Culiacán, Sinaloa. México.
3. Facultad de Biología, Universidad Autónoma de Sinaloa. Culiacán, Sinaloa, México.
4. Departamento de Anatomía Patológica, Centro de Investigación y Docencia en Ciencias de la Salud, Universidad Autónoma de Sinaloa, Hospital Civil de Culiacán, México.
5. Departamento de investigación del Hospital de la Mujer, Servicios de Salud de Sinaloa. Culiacán, Sinaloa, México.
6. Unidad de Investigación Biomédica de Zacatecas, Instituto Mexicano del Seguro Social, Zacatecas, Zacatecas, México.
7. Departamento de Ciencias Naturales, Universidad Autónoma Metropolitana, Unidad Cuajimalpa. Ciudad de México, México.
8. Departamento de Infectómica y Patogénesis Molecular. Centro de Investigación y de Estudios Avanzados del Instituto politécnico Nacional, Ciudad de México, México.
9. Departamento de Anatomía Patológica, Instituto Mexicano del Seguro Social. Culiacán, Sinaloa, México.
10. Departamento de Investigación en Salud, Unidad Médica de Alta Especialidad, Hospital de Especialidades No. 14, Centro Médico Nacional “Adolfo Ruiz Cortines”, Instituto Mexicano del Seguro Social (IMSS), Veracruz, México
11. Facultad de Medicina, Región Veracruz, Universidad Veracruzana (UV), Veracruz, México.

***Address correspondence to:**

Juan Fidel Osuna-Ramos (osunajuanfidel.fm@uas.edu.mx)

José Manuel Reyes-Ruiz (jose.reyesr@imss.gob.mx)

**Supplemental Material**

suppmat

2024-04-25

#required packages
library(tidyverse)

## ── Attaching core tidyverse packages ──────────────────────── tidyverse 2.0.0 ──
## ✔ dplyr 1.1.4 ✔ readr 2.1.4
## ✔ forcats 1.0.0 ✔ stringr 1.5.1
## ✔ ggplot2 3.4.4 ✔ tibble 3.2.1
## ✔ lubridate 1.9.3 ✔ tidyr 1.3.0
## ✔ purrr 1.0.2
## ── Conflicts ────────────────────────────────────────── tidyverse_conflicts() ──
## ✖ dplyr::filter() masks stats::filter()
## ✖ dplyr::lag() masks stats::lag()
## ℹ Use the conflicted package (<http://conflicted.r-lib.org/>) to force all conflicts to become errors

library(ggplot2)
library(Rcpp)
library(dlookr)

## Registered S3 methods overwritten by 'dlookr':
## method from
## plot.transform scales
## print.transform scales
##
## Attaching package: 'dlookr'
##
## The following object is masked from 'package:tidyr':
##
## extract
##
## The following object is masked from 'package:base':
##
## transform

library(ggpubr)
library(gt)
library(DescTools)
library(funModeling)

## Loading required package: Hmisc
##
## Attaching package: 'Hmisc'
##
## The following objects are masked from 'package:DescTools':
##
## %nin%, Label, Mean, Quantile
##
## The following object is masked from 'package:gt':
##
## html
##
## The following object is masked from 'package:dlookr':
##
## describe
##
## The following objects are masked from 'package:dplyr':
##
## src, summarize
##
## The following objects are masked from 'package:base':
##
## format.pval, units
##
## funModeling v.1.9.5 :)
## Examples and tutorials at livebook.datascienceheroes.com
## / Now in Spanish: librovivodecienciadedatos.ai

library(readxl)
covilipidos_survival_analysis <- read_excel("Stratified_Sample_Covilipidos_Survival_Analysis.xlsx")

covilipids <- covilipidos_survival_analysis

lipidb<-covilipids

# Creating new binary variables based on the cut-off points of Clinic predictor variable
lipidb$bin_cholclin <- ifelse(lipidb$Chol <= 99.5, 1, 0)
lipidb$bin_ldlcclin <- ifelse(lipidb$LDLc <= 48.5, 1, 0)
lipidb$bin_hdlcclin <- ifelse(lipidb$HDLc <= 25.5, 1, 0)
lipidb$bin_trygclin <- ifelse(lipidb$Tryg >= 139.5, 1, 0)

# Creating new binary variables based on the cut-off points of outcome predictor variable
lipidb$bin_cholout <- ifelse(lipidb$Chol >= 138.5, 1, 0)
lipidb$bin_ldlcout <- ifelse(lipidb$LDLc <= 44.5, 1, 0)
lipidb$bin_hdlcout <- ifelse(lipidb$HDLc <= 24.5, 1, 0)
lipidb$bin_trygout <- ifelse(lipidb$Tryg >= 156.5, 1, 0)


# Logistic regression for the clinic context
logit_model_clinic <- glm(Clinic ~Age +Sex +bin_cholclin + bin_ldlcclin + bin_hdlcclin + bin_trygclin + Diabetes + Hypertension + Obesity, family = "binomial", data = lipidb)

# Logistic regression for the outcome
logit_model_outcome <- glm(outcome ~ Age +Sex +bin_cholout + bin_ldlcout + bin_hdlcout + bin_trygout + Diabetes + Hypertension + Obesity, family = "binomial", data = lipidb)

library(car)

## Loading required package: carData

##
## Attaching package: 'car'

## The following object is masked from 'package:DescTools':
##
## Recode

## The following object is masked from 'package:dplyr':
##
## recode

## The following object is masked from 'package:purrr':
##
## some

# Calculate VIF for the clinic context model
vif_clinic <- vif(logit_model_clinic)
print(vif_clinic)

## Age Sex bin_cholclin bin_ldlcclin bin_hdlcclin bin_trygclin
## 1.021515 1.027284 1.010051 1.007432 1.019458 1.019823
## Diabetes Hypertension Obesity
## 1.016010 1.011916 1.027688

# Calculate VIF for the outcome model
vif_outcome <- vif(logit_model_outcome)
print(vif_outcome)

## Age Sex bin_cholout bin_ldlcout bin_hdlcout bin_trygout
## 1.016795 1.023721 1.012933 1.003763 1.020238 1.022436
## Diabetes Hypertension Obesity
## 1.015181 1.020691 1.027953

# Assuming logit_model_clinic and logit_model_outcome are your logistic regression models

# Generate predicted probabilities for the clinic model
predicted_probs_clinic <- predict(logit_model_clinic, type = "response")


# Generate predicted probabilities for the outcome model
predicted_probs_outcome <- predict(logit_model_outcome, type = "response")

predicted_probs_clinic

## 1 2 3 4 5 6 7 8
## 0.4986762 0.3948234 0.5411413 0.4437898 0.4201537 0.5353597 0.4919417 0.5473658
## 9 10 11 12 13 14 15 16
## 0.4298243 0.4658512 0.4687237 0.4811244 0.5677548 0.4955507 0.4883953 0.5384077
## 17 18 19 20 21 22 23 24
## 0.4759025 0.5622438 0.4730971 0.4955340 0.5384539 0.5997005 0.3422341 0.4915912
## 25 26 27 28 29 30 31 32
## 0.4492690 0.4704627 0.5376634 0.5510964 0.5033767 0.5117695 0.4355631 0.5086693
## 33 34 35 36 37 38 39 40
## 0.4825225 0.5293557 0.5168869 0.4187201 0.5702463 0.4844415 0.4445444 0.4579465
## 41 42 43 44 45 46 47 48
## 0.4175061 0.5799761 0.4536006 0.4882736 0.4836005 0.4820882 0.5120186 0.4146960
## 49 50 51 52 53 54 55 56
## 0.4323010 0.3568203 0.5130306 0.4687846 0.5203112 0.5225963 0.4238813 0.4236449
## 57 58 59 60 61 62 63 64
## 0.3774498 0.5364075 0.4673960 0.4817284 0.4263503 0.4991588 0.5155224 0.4525182
## 65 66 67 68 69 70 71 72
## 0.5112526 0.4558574 0.4626788 0.4937337 0.4495555 0.5257002 0.4095130 0.4745243
## 73 74 75 76 77 78 79 80
## 0.5537641 0.3887527 0.4170367 0.4909213 0.5666924 0.5255609 0.3635835 0.5330725
## 81 82 83 84 85 86 87 88
## 0.5377947 0.5134512 0.5565797 0.5128600 0.6099302 0.3066229 0.4762826 0.4613620
## 89 90 91 92 93 94 95 96
## 0.4519319 0.5139019 0.5769963 0.5200000 0.4321090 0.5549027 0.4065385 0.4545597
## 97 98 99 100 101 102 103 104
## 0.4262291 0.3236985 0.5092689 0.4928920 0.5601706 0.4291271 0.4365822 0.4788032
## 105 106 107 108 109 110 111 112
## 0.4908582 0.3733785 0.4973098 0.3631940 0.4898841 0.5090901 0.4649519 0.4880484
## 113 114 115 116 117 118 119 120
## 0.5029558 0.5113478 0.5217809 0.5388723 0.4785148 0.4544273 0.5490178 0.3484305
## 121 122 123 124 125 126 127 128
## 0.4307563 0.3875384 0.4881548 0.4658360 0.5116614 0.5480642 0.4521010 0.4596472
## 129 130 131 132 133 134 135 136
## 0.5180769 0.3889227 0.4553948 0.4610049 0.3871061 0.5123206 0.5014974 0.4953087
## 137 138 139 140 141 142 143 144
## 0.5306724 0.4191300 0.4738665 0.5871075 0.4315907 0.4651299 0.4758626 0.5597874
## 145 146 147 148 149 150 151 152
## 0.4354013 0.4688318 0.5338478 0.5726515 0.4207304 0.4919556 0.4542732 0.3939645
## 153 154 155 156 157 158 159 160
## 0.4989181 0.5591664 0.5033528 0.4982552 0.4425409 0.4568988 0.5591318 0.4082921
## 161 162 163 164 165 166 167 168
## 0.6242517 0.3934055 0.4421502 0.5051358 0.4890716 0.4790281 0.5840722 0.5190489
## 169 170 171 172 173 174 175 176
## 0.4576591 0.5158259 0.5042186 0.4065661 0.5824049 0.5579209 0.5604805 0.5398467
## 177 178 179 180 181 182 183 184
## 0.3907550 0.3185596 0.3873087 0.5451089 0.3181960 0.4843382 0.5696583 0.6089583
## 185 186 187 188 189 190 191 192
## 0.4275862 0.5217503 0.4930387 0.3667619 0.5623893 0.4229124 0.5455592 0.5891468
## 193 194 195 196 197 198 199 200
## 0.3698398 0.4861268 0.4002371 0.4456152 0.4688318 0.4699255 0.4754377 0.4821605
## 201 202 203 204 205 206 207 208
## 0.3984499 0.4013954 0.5287082 0.5203098 0.4016730 0.4527814 0.6069517 0.4543182
## 209 210 211 212 213 214 215 216
## 0.4999853 0.4989181 0.5285766 0.4476031 0.4575899 0.4807245 0.5280487 0.4632768
## 217 218 219 220 221 222 223 224
## 0.4575285 0.4819167 0.5477618 0.3915570 0.5222478 0.5311237 0.4915963 0.4876277
## 225 226 227 228 229 230 231 232
## 0.4909966 0.5678633 0.5783345 0.4671549 0.4068996 0.5101467 0.5625534 0.4201979
## 233 234 235 236 237 238 239 240
## 0.5257145 0.4304125 0.4264859 0.5671659 0.5473743 0.3283509 0.4204488 0.3678794
## 241 242 243 244 245 246 247 248
## 0.4575984 0.5639720 0.5887989 0.4567789 0.6110349 0.5341587 0.4073060 0.6290494
## 249 250 251 252 253 254 255 256
## 0.4513363 0.3881207 0.4578848 0.5286381 0.5585423 0.6057150 0.4163772 0.5504331
## 257 258 259 260 261 262 263 264
## 0.5552582 0.4028876 0.4512670 0.4911301 0.4204895 0.4159146 0.4998304 0.4613204
## 265 266 267 268 269 270 271 272
## 0.5018016 0.4316513 0.4568239 0.5015192 0.5184520 0.4285858 0.5126099 0.5019819
## 273 274 275 276 277 278 279 280
## 0.5231190 0.4815737 0.4923009 0.4998686 0.4664483 0.4733833 0.3935626 0.4694909
## 281 282 283 284 285 286 287 288
## 0.4586048 0.5046395 0.5123994 0.4881548 0.5053492 0.5144238 0.4179741 0.4612455
## 289 290 291 292 293 294 295 296
## 0.4832554 0.4452883 0.3614142 0.5355255 0.5100249 0.4782726 0.4081743 0.4203151
## 297 298 299 300 301 302 303 304
## 0.5447257 0.3531866 0.4708816 0.4059136 0.4630411 0.5860858 0.5055913 0.4839487
## 305 306 307 308 309 310 311 312
## 0.4648819 0.4596188 0.5048112 0.5909086 0.4885445 0.3823560 0.4630411 0.5100538
## 313 314 315 316 317 318 319 320
## 0.4692045 0.4672165 0.3644197 0.5676948 0.5083707 0.4616157 0.4852403 0.5343844
## 321 322 323 324 325 326 327 328
## 0.5861159 0.4053882 0.4008640 0.4058537 0.6272387 0.4071785 0.5933437 0.4059087
## 329 330 331 332 333 334 335 336
## 0.4190849 0.4138255 0.4510885 0.4446047 0.3901239 0.4920895 0.4652212 0.4920171
## 337 338 339 340 341 342 343 344
## 0.4845169 0.4275942 0.5189889 0.5243012 0.3585901 0.3917718 0.4062150 0.4957760
## 345 346 347 348 349 350 351 352
## 0.5163345 0.4308863 0.5157629 0.5432324 0.4096874 0.4427741 0.5154471 0.4597500
## 353 354 355 356 357 358 359 360
## 0.5295472 0.4380945 0.5477627 0.5840420 0.5248605 0.4170818 0.6300606 0.4503869
## 361 362 363 364 365 366 367 368
## 0.5652138 0.4301916 0.6014877 0.5966348 0.5331878 0.5654517 0.4693263 0.4698021
## 369 370 371 372 373 374 375 376
## 0.4889962 0.4426596 0.3790385 0.4258174 0.3267224 0.4469934 0.4589137 0.5060571
## 377 378 379 380 381 382 383 384
## 0.3905817 0.4124727 0.4234981 0.5104456 0.4581237 0.4629794 0.5067439 0.4774324
## 385 386 387 388 389 390 391 392
## 0.5404390 0.5376939 0.4655403 0.6039049 0.4672012 0.3766062 0.5809719 0.4063045
## 393 394 395 396 397 398 399 400
## 0.4865475 0.4563049 0.4522319 0.4306044 0.5179064 0.4283023 0.5419773 0.5235390
## 401 402 403 404 405 406 407 408
## 0.4410730 0.4996983 0.4730971 0.4089881 0.3784145 0.5047634 0.4537367 0.5419773
## 409 410 411 412 413 414 415 416
## 0.5419323 0.6001046 0.4550222 0.3253871 0.5360886 0.4709289 0.4003681 0.4996983
## 417 418 419 420 421 422 423 424
## 0.3988731 0.4001245 0.5018771 0.5705140 0.4655489 0.4723187 0.5805619 0.5652138
## 425 426 427 428 429 430 431 432
## 0.4922394 0.4490142 0.5171270 0.3411400 0.4639343 0.3889207 0.5220525 0.4645945
## 433 434 435 436 437 438 439 440
## 0.4536063 0.6026959 0.4730270 0.3815304 0.5211537 0.4456993 0.4727526 0.5429223
## 441 442 443 444 445 446 447 448
## 0.5711637 0.5852389 0.3399636 0.4894470 0.4971727 0.3865244 0.5389436 0.4216651
## 449 450 451 452 453 454 455 456
## 0.4934463 0.3644896 0.4636955 0.4226762 0.3504129 0.4213103 0.5511574 0.4751378
## 457 458 459 460 461 462 463 464
## 0.4663868 0.5341029 0.5179679 0.5156254 0.4438462 0.5486255 0.4661058 0.5952462
## 465 466 467 468 469 470 471 472
## 0.4694578 0.4990660 0.4517699 0.5693449 0.4221768 0.4461058 0.5670028 0.6070256
## 473 474 475 476 477 478 479 480
## 0.5124560 0.5088232 0.5561499 0.4601069 0.4626090 0.4178556 0.4261733 0.4778236
## 481 482 483 484 485 486 487 488
## 0.5207335 0.3604419 0.4995798 0.5515739 0.4346498 0.4698167 0.4298982 0.5580974
## 489 490 491 492 493 494 495 496
## 0.3966772 0.5742231 0.5076320 0.3602492 0.6010821 0.4292354 0.4282893 0.5782287
## 497 498 499 500
## 0.5359888 0.4534835 0.4288310 0.5508155

predicted_probs_outcome

## 1 2 3 4 5 6 7
## 0.14565922 0.13192188 0.09816535 0.15163866 0.38238945 0.16053051 0.13556623
## 8 9 10 11 12 13 14
## 0.26148289 0.16246054 0.07938129 0.10930196 0.16147323 0.21020763 0.11965891
## 15 16 17 18 19 20 21
## 0.12631679 0.26230152 0.13329407 0.15939878 0.09099454 0.21465647 0.11566810
## 22 23 24 25 26 27 28
## 0.13791953 0.15559726 0.19198725 0.13623961 0.07545564 0.12115115 0.09960681
## 29 30 31 32 33 34 35
## 0.09117175 0.20778964 0.15683587 0.09879429 0.23835034 0.19590629 0.18871566
## 36 37 38 39 40 41 42
## 0.18166611 0.14248338 0.06367073 0.10802771 0.13673020 0.12345074 0.13266455
## 43 44 45 46 47 48 49
## 0.20936784 0.15029807 0.08117489 0.13755551 0.17478432 0.18016488 0.07164896
## 50 51 52 53 54 55 56
## 0.27423338 0.07609242 0.30975897 0.17643631 0.13928405 0.07177401 0.06247714
## 57 58 59 60 61 62 63
## 0.24148798 0.12275655 0.14136781 0.24801791 0.06980180 0.12896115 0.16638830
## 64 65 66 67 68 69 70
## 0.09549415 0.28793698 0.09209626 0.07145916 0.14152647 0.05367699 0.13038124
## 71 72 73 74 75 76 77
## 0.11775274 0.14566594 0.17476611 0.07793049 0.13954963 0.12557192 0.14588492
## 78 79 80 81 82 83 84
## 0.17914287 0.09629344 0.18167627 0.10176206 0.07574183 0.16384451 0.17334737
## 85 86 87 88 89 90 91
## 0.18278648 0.12195954 0.14629066 0.10325028 0.25788456 0.16231137 0.14816829
## 92 93 94 95 96 97 98
## 0.08106708 0.12334574 0.23708084 0.11563449 0.18787656 0.16622474 0.16865813
## 99 100 101 102 103 104 105
## 0.08553790 0.14274520 0.16277545 0.18184022 0.24169160 0.25913306 0.13850146
## 106 107 108 109 110 111 112
## 0.08595604 0.20117253 0.12106858 0.25380339 0.14866413 0.09073688 0.08884842
## 113 114 115 116 117 118 119
## 0.11479653 0.07750974 0.22125448 0.11515789 0.12303083 0.16123709 0.12221940
## 120 121 122 123 124 125 126
## 0.17695296 0.20943481 0.15007535 0.14092400 0.24012838 0.09946905 0.13265835
## 127 128 129 130 131 132 133
## 0.09592668 0.14942117 0.07198209 0.08527585 0.18635628 0.07279698 0.23406571
## 134 135 136 137 138 139 140
## 0.18710279 0.19354254 0.10486020 0.10979472 0.18092434 0.07563431 0.15739300
## 141 142 143 144 145 146 147
## 0.15741472 0.12049174 0.14958902 0.25882516 0.14091668 0.12210418 0.12141111
## 148 149 150 151 152 153 154
## 0.21515626 0.23735513 0.19382019 0.12933509 0.12763300 0.11551099 0.11029216
## 155 156 157 158 159 160 161
## 0.22472330 0.14628223 0.19009586 0.13495378 0.35846011 0.14429376 0.15742776
## 162 163 164 165 166 167 168
## 0.17190085 0.13542447 0.15673445 0.23487972 0.28383019 0.12701898 0.17524419
## 169 170 171 172 173 174 175
## 0.21575594 0.16813913 0.09034694 0.19149851 0.14294332 0.11177197 0.10313570
## 176 177 178 179 180 181 182
## 0.27922283 0.07615378 0.17869679 0.16370155 0.22757034 0.10584302 0.08396699
## 183 184 185 186 187 188 189
## 0.16299201 0.20851475 0.08682973 0.18883445 0.36251111 0.10979179 0.15448327
## 190 191 192 193 194 195 196
## 0.16243736 0.10725752 0.15410742 0.08955627 0.24122471 0.10883226 0.12213620
## 197 198 199 200 201 202 203
## 0.10051909 0.16444237 0.08094256 0.09467774 0.12685582 0.10287394 0.15924583
## 204 205 206 207 208 209 210
## 0.17308779 0.12191002 0.11501118 0.21266866 0.09860866 0.23176533 0.17292029
## 211 212 213 214 215 216 217
## 0.18730364 0.10379783 0.11617720 0.15878024 0.14377032 0.17330982 0.11339353
## 218 219 220 221 222 223 224
## 0.14273225 0.10325028 0.07545355 0.15559129 0.17987452 0.18805442 0.07079525
## 225 226 227 228 229 230 231
## 0.21348937 0.10040719 0.13498165 0.08141781 0.11086141 0.20039094 0.10084718
## 232 233 234 235 236 237 238
## 0.26170887 0.11816562 0.20045641 0.22492366 0.23776147 0.24494100 0.18146550
## 239 240 241 242 243 244 245
## 0.16655829 0.25348107 0.08561429 0.08654857 0.13777879 0.15642416 0.20817904
## 246 247 248 249 250 251 252
## 0.15073883 0.11036976 0.24959173 0.23626155 0.11912173 0.25787883 0.16745898
## 253 254 255 256 257 258 259
## 0.26171164 0.16577381 0.18351945 0.18060735 0.09521358 0.14960516 0.09679701
## 260 261 262 263 264 265 266
## 0.45569502 0.11787583 0.13037784 0.10853963 0.14689831 0.12371311 0.19806428
## 267 268 269 270 271 272 273
## 0.07624305 0.12778339 0.11878943 0.05905526 0.07644450 0.11024953 0.19739457
## 274 275 276 277 278 279 280
## 0.11747234 0.12204006 0.22211686 0.06853169 0.13679641 0.12819047 0.08925577
## 281 282 283 284 285 286 287
## 0.10251369 0.11278076 0.19422683 0.11310662 0.13280088 0.18333196 0.29766690
## 288 289 290 291 292 293 294
## 0.10488824 0.11541571 0.14860864 0.08605337 0.06831632 0.23465811 0.34366097
## 295 296 297 298 299 300 301
## 0.11415445 0.10805413 0.18111004 0.31548789 0.21470269 0.22720613 0.14974661
## 302 303 304 305 306 307 308
## 0.08884842 0.12137350 0.14708348 0.11996019 0.16645670 0.10855277 0.22755341
## 309 310 311 312 313 314 315
## 0.12478689 0.15855191 0.14974661 0.17559588 0.07650824 0.07777896 0.23662521
## 316 317 318 319 320 321 322
## 0.08305822 0.17850848 0.31280868 0.30363409 0.29768841 0.12427394 0.11110043
## 323 324 325 326 327 328 329
## 0.12298402 0.09791007 0.17310380 0.13115943 0.12466883 0.10222986 0.10950761
## 330 331 332 333 334 335 336
## 0.07615588 0.10881870 0.26246044 0.08411362 0.13127090 0.19779800 0.18729254
## 337 338 339 340 341 342 343
## 0.11389384 0.17191977 0.18491972 0.14692439 0.25043217 0.15996125 0.24313406
## 344 345 346 347 348 349 350
## 0.24078244 0.22146386 0.06631576 0.18452670 0.16211974 0.12122960 0.23168798
## 351 352 353 354 355 356 357
## 0.09551212 0.07381545 0.15791218 0.15850306 0.26536828 0.14051176 0.13151869
## 358 359 360 361 362 363 364
## 0.22559518 0.27645828 0.09398892 0.08537061 0.09702608 0.11853746 0.12494827
## 365 366 367 368 369 370 371
## 0.10689808 0.09771906 0.17367599 0.06602315 0.11210785 0.05029113 0.32750133
## 372 373 374 375 376 377 378
## 0.16691853 0.16628405 0.27495516 0.11415990 0.19434503 0.21652963 0.12393165
## 379 380 381 382 383 384 385
## 0.14198668 0.23376178 0.18115221 0.38217636 0.08791271 0.39694517 0.14423371
## 386 387 388 389 390 391 392
## 0.11908116 0.10677707 0.19425137 0.19430400 0.14587210 0.16759177 0.22710907
## 393 394 395 396 397 398 399
## 0.24031117 0.24993812 0.08020611 0.09658912 0.16492132 0.11159790 0.09728404
## 400 401 402 403 404 405 406
## 0.14975971 0.11609845 0.12903457 0.07220451 0.11314761 0.35173058 0.21576877
## 407 408 409 410 411 412 413
## 0.39821367 0.09728404 0.23156208 0.13732641 0.11644322 0.18747979 0.22398331
## 414 415 416 417 418 419 420
## 0.07811668 0.46593597 0.12903457 0.12356780 0.11054785 0.21064265 0.14645765
## 421 422 423 424 425 426 427
## 0.07846751 0.18762970 0.16829011 0.08537061 0.12664789 0.26463605 0.19739932
## 428 429 430 431 432 433 434
## 0.20067672 0.20334214 0.11807692 0.30171660 0.08048388 0.19299969 0.19660874
## 435 436 437 438 439 440 441
## 0.11684746 0.15068366 0.12868470 0.12394536 0.12879681 0.13091910 0.16534715
## 442 443 444 445 446 447 448
## 0.14106744 0.15957772 0.34790293 0.16413477 0.08764408 0.15942667 0.34261709
## 449 450 451 452 453 454 455
## 0.12231624 0.36944478 0.09198144 0.14320871 0.18152746 0.09326094 0.08804693
## 456 457 458 459 460 461 462
## 0.22697443 0.07774780 0.10814143 0.15917478 0.19102177 0.19433658 0.24217881
## 463 464 465 466 467 468 469
## 0.20301262 0.14458670 0.07589181 0.11176893 0.13274934 0.14090757 0.15462658
## 470 471 472 473 474 475 476
## 0.20739826 0.13113591 0.21653715 0.09486138 0.07968108 0.23437977 0.08329589
## 477 478 479 480 481 482 483
## 0.08181479 0.11097820 0.19641702 0.37063374 0.10345624 0.14198725 0.12840081
## 484 485 486 487 488 489 490
## 0.08764648 0.28632939 0.32447397 0.26828111 0.09740003 0.19449313 0.14092400
## 491 492 493 494 495 496 497
## 0.20612691 0.10948088 0.19978541 0.06756429 0.16279042 0.21526796 0.12329574
## 498 499 500
## 0.12089643 0.13718949 0.15498201

# Load necessary library
library(pROC)

## Type 'citation("pROC")' for a citation.

##
## Attaching package: 'pROC'

## The following objects are masked from 'package:stats':
##
## cov, smooth, var

# Create ROC curve for the clinic model
roc_curve_clinic <- roc(response = lipidb$Clinic, # Replace 'your_data_frame$Clinic' with your actual data column
 predictor = predicted_probs_clinic)

## Setting levels: control = 0, case = 1

## Setting direction: controls < cases

# Create ROC curve for the outcome model
roc_curve_outcome <- roc(response = lipidb$outcome, # Replace 'your_data_frame$Outcome' with your actual data column
 predictor = predicted_probs_outcome)

## Setting levels: control = 0, case = 1
## Setting direction: controls < cases

# Plot ROC curve for the clinic model
plot(roc_curve_clinic, main = "ROC Curve for Clinic Model")


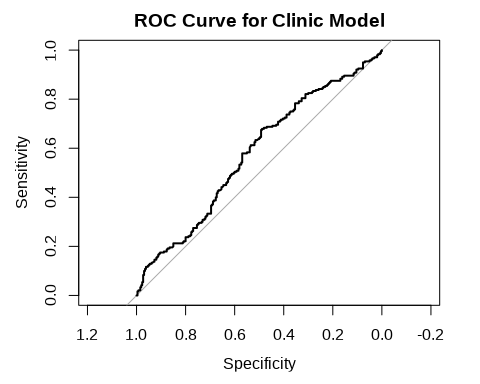


# Plot ROC curve for the outcome model
plot(roc_curve_outcome, main = "ROC Curve for Outcome Model")


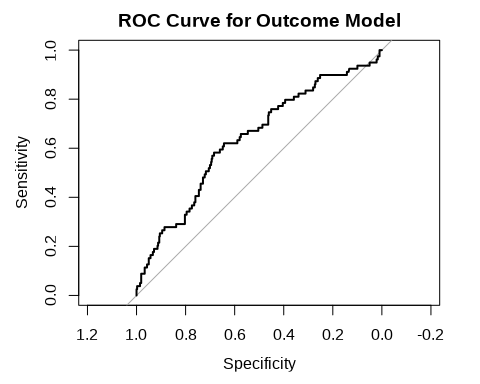


# Calculate and print AUC for both models
auc_clinic <- auc(roc_curve_clinic)
auc_outcome <- auc(roc_curve_outcome)
print(paste("AUC for Clinic Model:", auc_clinic))

## [1] "AUC for Clinic Model: 0.575817307692308"

print(paste("AUC for Outcome Model:", auc_outcome))

## [1] "AUC for Outcome Model: 0.637466550407409"

## K FOLD meand and

#install.packages("caret")

library(caret)

## Loading required package: lattice

##
## Attaching package: 'caret'

## The following objects are masked from 'package:DescTools':
##
## MAE, RMSE

## The following object is masked from 'package:purrr':
##
## lift

# Assuming your data frame is named 'lipidb' and the outcome variable is binary (0 or 1)
set.seed(123) # Setting seed for reproducibility
folds <- createFolds(lipidb$outcome, k = 1000) # Creates a list of 10 folds

# Convert the outcome variable to a factor with two levels
# Using more descriptive level names such as "Level0" and "Level1" or "Class0" and "Class1"

lipidb$outcome <- factor(lipidb$outcome, levels = c(0, 1), labels = c("Class0", "Class1"))


# Check the structure to ensure it's now a factor with valid names
str(lipidb$outcome)

## Factor w/ 2 levels "Class0","Class1": 1 2 1 2 1 1 1 1 1 1 ...

# Setting up the trainControl object with ROC metric and classProbs = TRUE
train_control <- trainControl(method = "cv",
 number = 100,
 summaryFunction = twoClassSummary,
 classProbs = TRUE, # Save class probabilities
 savePredictions = "final") # Save predictions for each fold


# Fit the logistic regression model with cross-validation
logistic_model_cv <- train(outcome ~ .,
 data = lipidb,
 method = "glm",
 family = binomial(),
 trControl = train_control,
 metric = "ROC")

## Warning in nominalTrainWorkflow(x = x, y = y, wts = weights, info = trainInfo,
## : There were missing values in resampled performance measures.

# Print the results
print(logistic_model_cv)

## Generalized Linear Model
##
## 500 samples
## 27 predictor
## 2 classes: 'Class0', 'Class1'
##
## No pre-processing
## Resampling: Cross-Validated (100 fold)
## Summary of sample sizes: 495, 495, 494, 495, 495, 495, ...
## Resampling results:
##
## ROC Sens Spec
## 0.5753165 0.9855 0.01265823

# Check the performance results
results <- logistic_model_cv$results
print(results)

## parameter ROC Sens Spec ROCSD SensSD SpecSD
## 1 none 0.5753165 0.9855 0.01265823 0.3657512 0.05786391 0.1125088

# Examine saved predictions and calculate ROC curve if needed
predictions <- logistic_model_cv$pred
